# p53 as a Regulator of Lipid Metabolism in Cancer

**DOI:** 10.3390/ijms17122074

**Published:** 2016-12-10

**Authors:** Alejandro Parrales, Tomoo Iwakuma

**Affiliations:** Department of Cancer Biology, University of Kansas Medical Center, Kansas City, KS 66160, USA; aparrales@kumc.edu

**Keywords:** p53, lipid metabolism, cancer, mevalonate pathway, fatty acid oxidation

## Abstract

Enhanced proliferation and survival are common features of cancer cells. Cancer cells are metabolically reprogrammed which aids in their survival in nutrient-poor environments. Indeed, changes in metabolism of glucose and glutamine are essential for tumor progression. Thus, metabolic reprogramming is now well accepted as a hallmark of cancer. Recent findings suggest that reprogramming of lipid metabolism also occurs in cancer cells, since lipids are used for biosynthesis of membranes, post-translational modifications, second messengers for signal transduction, and as a source of energy during nutrient deprivation. The tumor suppressor p53 is a transcription factor that controls the expression of proteins involved in cell cycle arrest, DNA repair, apoptosis, and senescence. p53 also regulates cellular metabolism, which appears to play a key role in its tumor suppressive activities. In this review article, we summarize non-canonical functions of wild-type and mutant p53 on lipid metabolism and discuss their association with cancer progression.

## 1. Introduction

Cancer cells have an extraordinary ability to adapt themselves to adverse environments. In other words, cancers can change their characteristics by modifying pathways or altering protein expression so that they can survive and expand in various environments. Cancer cells usually reside in poor oxygen and nutrition environments, and hence attempt to reprogram cellular metabolism [1].

There are common modifications in metabolic pathways that are well correlated with cancer progression [2]. Warburg et al. [3] first observed that cancer cells exhibit an increase in glucose uptake and glycolysis as an adaptive event in nutrient deprived conditions. This leads to enhanced glucose dependence and increased lactate production in cancer cells, also referred to as the Warburg effect. However, glucose is not the only biomolecule on which cancer cells rely. Glutamine consumption is also high in cancer cells, likely due to an increase in demand of carbon structures and amino-nitrogen which are required for nucleotide, protein, and lipid synthesis [4]. Besides these changes in glucose and amino acid metabolism, fast-proliferating cancer cells also have high avidity for lipids (fatty acids and cholesterol) which are mainly used for biosynthesis of structural components of the cells (membranes), as well as for production of energy during nutrient deprivation [5]. In physiological conditions, lipid synthesis is restricted to specialized tissues, such as liver and adipose tissues, while normal cells from other tissues obtain lipids directly from the bloodstream [5]. On the other hand, cancer cells often gain the ability to synthesize lipids and show enhanced lipid uptake [6]. Several studies have shown that upregulation of enzymes involved in the synthesis of fatty acids and cholesterol (mevalonate pathway) is required for tumor progression [5,6,7,8]. Specifically, a wide variety of tumors have increased expression of acetyl-CoA-carboxylase (ACC) [6] that catalyzes the conversion of acetyl-CoA to malonyl-CoA, the rate-limiting step in the lipid biosynthesis [5]. Another enzyme involved in lipid metabolism and highly overexpressed in cancers is fatty acid synthase (FAS), an enzyme that converts acetyl-CoA to palmitic acid [9]. High expression of this enzyme is also correlated with poor prognosis in cancer patients [9]. Additionally, tumors manifest an increase in the expression of ATP citrate lyase (ACLY) which catalyzes the conversion of citrate to oxaloacetic acid and acetyl-CoA, as well as an increase in transcriptional activities of the sterol regulatory element binding proteins (SREBPs); both ACLY and SREBPs promote cholesterol production [10].

Tumor suppressor p53 is the most frequently mutated human gene in cancer [11]. p53 suppresses tumorigenesis by regulating transcription of numerous downstream target genes that govern cell cycle progression and cell death [12]. p53 also regulates the expression of metabolism-associated proteins, and some of them play key roles in tumor suppression [13,14,15]. Previous reports have shown that both wild-type and mutant p53 are involved in the glucose metabolism and nucleotide synthesis by regulating levels or activities of glucose-6-phosphate dehydrogenase (G6PD) [16]; TP53-induced glycolysis and the apoptosis regulator (TIGAR) [17]; glucose transporter 1 (GLUT1) [18]; deoxycytidine kinase (dCK); and many other nucleotide metabolism-related proteins [19], which is described in details elsewhere. In this review article, we summarize studies that focus on the contributions of both wild-type and mutant p53 to lipid metabolism.

## 2. Regulation of Lipid Metabolism by Wild-Type p53

There is an increasing number of studies showing that p53 regulates lipid metabolism by transcriptional control or protein–protein interaction. The following are effectors associated with lipid metabolism whose activities or levels are regulated by p53 (Table 1).

### 2.1. Glucose-6-Phosphate Dehydrogenase (G6PD)

**Figure 1:**
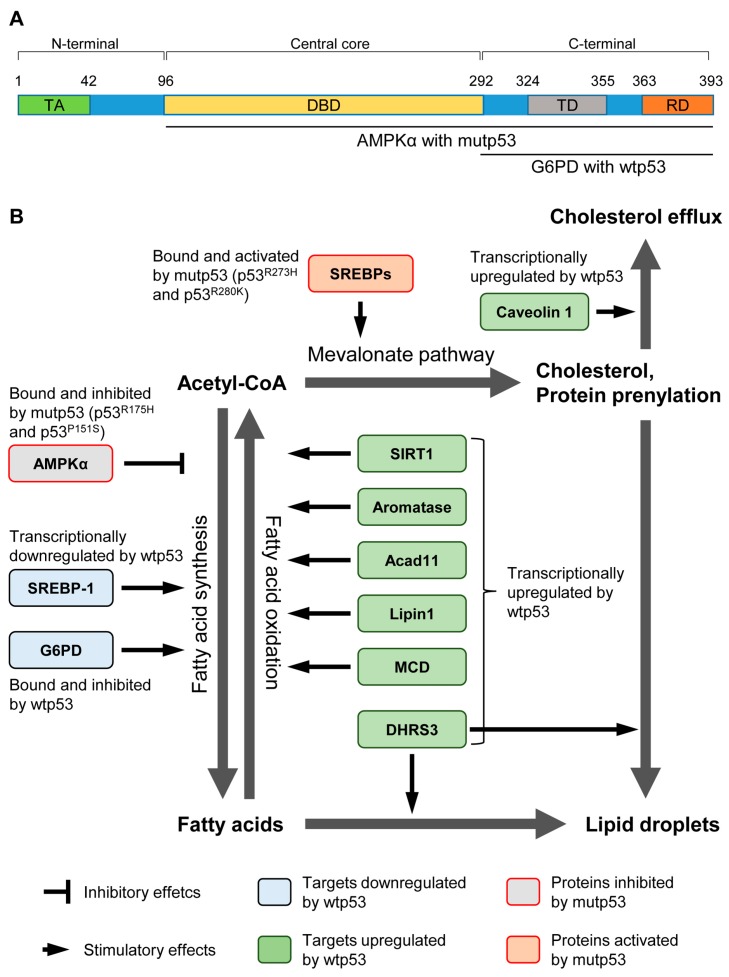
Regulation of lipid metabolism by wild-type and mutant p53. (**A**) Schematic representation of functional domains in p53 and regions which interact with G6PD and AMPKα. TA: transactivation domain, DBD: DNA-binding domain, TD: tetramerization domain, RD: regulatory domain; (**B**) Wild-type p53 (wtp53) can regulate lipid metabolism by direct protein–protein interaction or transcriptional control of proteins involved in fatty acid synthesis, fatty acid oxidation, the mevalonate pathway, cholesterol efflux, and lipid droplet formation. Generally, wtp53 inhibits the fatty acid synthesis and lipid accumulation. In contrast, mutant p53 (mutp53) enhances fatty acid synthesis by inhibitory interaction with AMPKα. Also, mutp53 cooperates with SREBPs to upregulate enzymes involved in the mevalonate pathway.

G6PD is a rate-limiting enzyme that catalyzes the first step in the pentose phosphate pathway (PPP). G6PD activation also increases NADPH production, which is required for lipid biosynthesis (Table 1) [29,30]. Interestingly, the carboxy (C)-terminal region of wild-type p53 directly binds with G6PD and inhibits its function (Figure 1A) [16]. Also, G6PD activity is increased in mouse embryonic fibroblasts (MEFs) and several tissues from *p53^−/−^* mice compared with those from wild-type mice [16]. Moreover, *p53^−/−^* MEFs and *p53^−/−^* colorectal carcinoma cell line HCT116 have increase in glucose uptake, PPP influx, and lipid accumulation, as compared with their counterparts having wild-type p53. A lack of p53 also results in a G6PD-dependent increase in NADPH in HCT116 cells [16]. These observations suggest that wild-type p53 reduces production of NADPH and inhibits accumulation of lipids by its direct binding to G6PD (Figure 1B). Importantly, an elevated expression of G6PD is correlated with poor clinical prognosis in esophageal squamous cell carcinoma [31]. Given that enhanced lipid biosynthesis is a common feature of cancer cells, inhibition of G6PD activity by p53 could contribute to p53-mediated tumor suppression.

### 2.2. Sterol Regulatory Element-Binding Protein-1 (SREBP-1)

SREBPs are a family of basic helix–loop–helix leucine zipper transcription factors that control the expression of a range of lipogenic enzymes required for the synthesis of cholesterol, fatty acid, triacylglycerol, and phospholipid (Table 1) [32]. Specifically, SREBP-1, but not SREBP-2, is shown to be well correlated with fatty acids synthesis induced by refeeding following fasting in mice [33]. SREBP-1 is frequently upregulated in multiple types of cancer, including glioblastoma and prostate cancer, and contributes towards tumor progression [34,35]. Also, levels of SREBP-1 are found to be negatively correlated with p53 levels in mice when fasting followed by refeeding [20]. Interestingly, *ob*/*ob* mice show reduced levels of SREBP-1 and its target enzymes with increase in p53 levels [20]. Moreover, *p53* deletion in *ob*/*ob* mice partially restores the levels of SREBP-1 and its downstream targets, including fatty acid synthase (FAS) [20]. Mechanistically, the exogenous expression of p53 in p53-null Saos2 osteosarcoma cells reduces the promoter activity of the *SREBP-1c* gene (Figure 1B) [20]. However, it still remains unclear how significantly a decrease in SREBP-1 levels contributes to p53-mediated tumor suppression.

### 2.3. Sirtuin 1 (SIRT1)

SIRT1 is an evolutionarily conserved NAD^+^-dependent protein deacetylase that targets proteins involved in fat cell maturation and accumulation, nutrient sensing, and regulation of cellular metabolism [36]. In hepatocyte-specific *SIRT1* knockout mice fed a high fat diet (HFD), there is a decrease in PPARα signaling and fatty acid β-oxidation, leading to an accumulation of fatty acids in the liver and hepatic steatosis (Figure 1B) [37]. Also, knockdown of SIRT1 leads to an increase in acetylation and activities of the liver X receptor (LXR) and SREBP-1 transcription factors, both of which contribute to the accumulation of lipids [38,39,40].

Interestingly, SIRT1 mRNA expression is induced by a complex of p53 and the forkhead transcription factor Foxo3a [21]. Upon nutrient starvation, p53 binds with Foxo3a, and this complex transactivates SIRT1 through two p53-responsive elements in its promoter (Table 1) [21]. In *p53^−/−^* mice, nutrient starvation fails to increase the SIRT1 expression [21]. Although *p53^−/−^* mice show higher levels of lipid accumulation when fed a HFD as compared with wild-type mice [22], it remains unclear whether this is caused by attenuation in SIRT1 levels in *p53^−/−^* mice.

### 2.4. Aromatase

Aromatase is the enzyme that catalyzes the final step in the biosynthesis of estrogens and is encoded by the *Cyp19* gene. Disruption of *Cyp19* in mice leads to increased adipocyte volume, hyperleptinemia, hyperinsulinemia, and hypercholesterolemia, when compared to wild-type mice, indicating that aromatase plays a key role in lipid metabolism [41]. A recent study from Wang et al. [22] shows that conversion of testosterone by aromatase is impaired in *p53^−/−^* mice, since *p53^−/−^* mice have higher levels of testosterone and lower levels of β-estradiol in the blood than wild-type mice when fed a HFD. They also show that p53 can transcriptionally upregulate aromatase [22]. Moreover, increased lipid accumulation in the livers of *p53^−/−^* mice is substantially nullified by transgenic expression of aromatase [22]. Given that *Cyp19* (aromatase) knockout mice show accumulation of lipid droplets in the liver [22,41], these observations strongly indicate that reduced aromatase activity following loss of p53 is at least partially responsible for the lipid accumulation in the *p53^−/−^* livers (Table 1). Thus, p53 regulates lipid metabolism in an aromatase-dependent manner (Figure 1B).

### 2.5. Acyl-CoA Dehydrogenase Family Member 11 (Acad11)

Acad11 belongs to a family of enzymes that catalyze the first step in every cycle of fatty acid β-oxidation in mitochondria [42]. Acad11 is shown to be a p53 target and contributes to pro-survival function of p53 during glucose starvation (Figure 1B) [23]. Expression of Acad11 is significantly higher following doxorubicin treatment in wild-type MEFs than that in *p53^−/−^* MEFs, due to the binding of p53 to its responsive elements present in introns of the *Acad11* gene [23]. Intriguingly, upon glucose starvation in MEFs expressing HRasV12, p53 promotes oxidative phosphorylation (OXPHOS) and cell survival in a manner dependent on Acad11 [23]. However, it is unclear whether increased Acad11 levels by p53 enhance fatty acid β-oxidation and how enhanced fatty acid β-oxidation contributes to cell survival under low glucose conditions (Table 1).

### 2.6. Lipin 1

Lipin 1 is a nuclear protein that is required for normal adipose tissue development [43]. Lipin 1 upregulates PPARα and activates the mitochondrial fatty acid oxidative metabolism (Table 1) [44]. Assaily et al. [24] show that p53 can upregulate Lipin 1 mRNA levels through three p53 binding sites in intron 1. Also, γ-irradiation in wild-type mice, but not in *p53^−/−^* mice, upregulates Lipin 1 mRNA levels in the spleen, thymus, and bone morrow [24]. Additionally, glucose restriction in mouse myoblast C2C12 cells phosphorylates p53 at Ser18 (Ser15 in humans), leading to the upregulation of Lipin 1 and fatty acid oxidation (Table 1) [24]. These observations indicate that increased Lipin 1 by p53 upon glucose deprivation enhances fatty acid oxidation (Figure 1B) [24]. However, it still remains unknown whether Lipin 1 plays a role in p53-mediated tumor suppression.

### 2.7. Malonyl-CoA Decarboxylase (MCD)

MCD that catalyzes the conversion of malonyl-CoA to acetyl-CoA is a key enzyme in lipid metabolism and enhances fatty acid oxidation, hence preventing fatty acids synthesis and lipid accumulation [45]. Importantly, a recent study using an inducible p53ER^TAM^ MEF cell system identifies MCD as a p53 target; activation of p53 via Mdm2 inhibition with nutlin-3 significantly increases MCD mRNA levels in wild-type, but not *p53^−/−^*, MEFs through p53’s binding to the promoter and intron regions of the *MCD* gene [25]. Liu et al. [25] also generate mice having a cancer-associated mutation in the *Mdm2* gene at codon 305 (Mdm2^C305F^) which disrupts the interaction of Mdm2 with ribosomal proteins (RPs), RPL11 and RPL5; during ribosomal stress, these RPs bind to Mdm2 and inhibit Mdm2’s function towards p53, hence activating p53 [46]. Interestingly, under ribosomal stress by fasting, *Mdm2^C305F^* mice show attenuated MCD induction, increase in malonyl-CoA, and increased fatty acid accumulation in the liver, due to the failure of RPs to bind with Mdm2 and the subsequent reduction in the p53 activity, as compared with mice having wild-type Mdm2 (Table 1) [25]. Thus, p53 inhibits lipid accumulation in the liver by transactivating MCD (Figure 1B). However, the contribution of MCD to p53-mediated tumor suppression needs to be investigated.

### 2.8. Dehydrogenase/Reductase 3 (DHRS3)

DHRS3, also known as retinal short-chain dehydrogenase/reductase (retSDR1), is a highly conserved member of the short chain alcohol dehydrogenase/reductase superfamily and is involved in maintaining the cellular supply of retinol metabolites [47]. DHSRS3 is localized in the endoplasmic reticulum (ER) and lipid droplets, both of which are storage vesicles containing lipid, such as cholesterol and retinol esters [48]. Additionally, overexpression of DHRS3 in U2OS cells increases lipid droplet accumulation (Figure 1B) [26]. Doxorubicin treatment is shown to increase the *DHRS3* mRNA expression in HCT116 cells having wild-type p53, but not HCT116 p53-null cells (Table 1) [27]. Indeed, there are two p53 responsible elements in the *DHRS3* promoter (Figure 1B) [27]. Deisenroth et al. [26] also show that overexpression of p53 in 3T3-L1 preadipocytes increases lipid droplet formation and the *DHRS3* mRNA expression is increased upon p53 induction in MEFs expressing p53ER^TAM^ in the absence of functional MDM2 (Mdm2-null or Mdm2^C462A^). Thus, p53 is involved in the accumulation of lipid droplets. However, it remains unclear whether p53-induced lipid droplet accumulation contributes to tumor suppression.

### 2.9. Caveolin 1

Caveolin 1 is the main component of the caveolae plasma membranes present in many different cell types [49]. Caveolin 1 is a scaffolding protein which binds to free cholesterol and is implicated in free cholesterol efflux [50]. Bist et al. [28] report that p53 binds to the promoter of *caveolin 1* in human skin fibroblasts. Overexpression of wild-type p53 results in an increase in the *caveolin 1* promoter activity and a decrease in intracellular free cholesterol in human skin fibroblasts, whereas mutant p53 fails to do so, suggesting that p53 increases cholesterol efflux, possibly through the upregulation of caveolin 1 (Figure 1B) [28]. Reduced intracellular free cholesterol through p53-induced caveolin 1 is correlated with decreased viable cell growth of human skin fibroblasts (Table 1) [28]. In line with this study, overexpression of caveolin 1 is shown to increase cholesterol efflux and reduce the proliferation of human skin fibroblasts [51]. However, dysregulation of caveolin 1 expression is found in multiple types of cancer [52,53,54]. It should be clarified in which cellular context caveolin 1 functions as a tumor suppressor or an oncogene and whether altered intracellular cholesterol levels by caveolin 1 could suppress or promote cancer progression.

## 3. Roles of Mutant p53 in the Lipid Metabolism 

Mutations in p53 are common events in the majority of cancers. Increasing evidence has demonstrated that many p53 mutants contribute to cancer progression by altering activities of other transcription factors, such as p63, p73, Ets2, and Nrf2, as their gain-of-function (GOF) activities [55,56,57]. Intriguingly, p53^R273H^ and p53^R280K^ GOF mutants are shown to bind with SREBPs, leading to their activation that causes the upregulation of enzymes involved in the mevalonate pathway (Figure 1B) [58]. This leads to an increase in cholesterol synthesis and protein prenylation (farnesylation and geranylgeranylation), which accelerates growth in the 3-dimensional culture of breast cancer cells (Table 2) [58]. Also, the presence of mutations in p53 correlates with high levels of enzymes involved in the mevalonate pathway in human breast cancer tissues [58]. These observations suggest the possibility that mutant p53 contributes to breast cancer progression through the upregulation of cholesterol production and protein prenylation.

A recent study has identified AMP-activated protein kinase (AMPK) as a novel binding partner of mutant p53 [59]. AMPK is a heterotrimeric protein kinase and is activated by low nutrient or energy levels [60]. Activated/phosphorylated AMPK inhibits fatty acid synthesis by phosphorylation and inhibition of both acetyl-CoA-carboxylase (ACC) and SREBP-1 (Table 2), thus linking availability of nutrients with lipid metabolism [61,62]. Zhou et al. [59] show that the ectopic expression of p53 GOF mutants (p53^R175H^ and p53^P151S^) inhibits AMPK activity and subsequently reduces phosphorylation of ACC under glucose and serum starvation in a p53-null head and neck squamous cell carcinoma (HNSCC) cell line, UMSCC1. AMPK activity is also reduced in MEFs expressing p53^R172H^ (equivalent with human p53^R175H^), but not in *p53^−/−^* MEFs [59]. In HNSCC mouse models, mutant p53 levels in tumors are negatively correlated with phosphorylation of AMPK [59]. Moreover, the downregulation of mutant p53 in Tu138 (p53^P151S^) and SK-Br-3 (p53^R175H^) cell lines increases AMPK activity upon energy stress induced by inhibition of GLUT1, one of the glucose transporters [59]. Thus, mutant p53 inhibits the activity of AMPK. The observed inhibition of AMPK activity is caused by the interaction of AMPK subunit α (AMPKα) with mutant p53 (p53^R175H^ and p53^P151S^) at the central core DNA-binding domain and C-terminal region (Figure 1A) [59]. Thus, p53 GOF mutants enhance not only tumor progression, but could also increase fatty acid synthesis by inhibiting AMPK (Figure 1B). It remains unclear how significantly the inhibitory effect of mutant p53 on AMPK activity contributes to the fatty acid synthesis and tumor progression.

## 4. Summary and Future Perspectives

In this review article, we focus on lipid metabolism pathways that are directly associated with p53, including fatty acid synthesis, fatty acid oxidation, cholesterol synthesis, cholesterol efflux, and lipid droplet formation. Other pathways in lipid metabolism include the synthesis of phospholipids, triglycerides, and ceramides [6]. However, to the best of our knowledge, there is no direct evidence showing the involvement of p53 in these processes.

Interestingly, recent data from our laboratory have demonstrated that mevalonte-5-phosphate (MVP), an intermediate metabolite in the mevalonate pathway, is involved in the stabilization of conformational p53 mutants, since the inhibition of MVP production leads to the degradation of conformational mutant p53 [63]. Given that stabilization of mutant p53 is required for its GOF activities [11,58,64,65,66,67], our study reveals a novel mechanism by which the mevalonate pathway contributes to cancer progression. Thus, the mevalonate pathway promotes tumorigenesis by two mechanisms: (1) by enhancing protein prenylation, which is required for the activation of proteins involved in proliferation and migration, such as Ras and Rho; and (2) by stabilizing conformational mutant p53. It should be noted that the efficacy of statins (cholesterol lowering drugs) that inhibit the mevalonate pathway on tumor suppression is promising, yet they still remain controversial [68,69,70,71,72]. Our study may suggest the importance of knowledge of the p53 status in tumors treated with statins to determine their efficacy.

Inhibition of fatty acid synthesis is shown to induce apoptosis in a pancreatic cancer cell line [73]. On the other hand, in a xenograft mouse model of myc-overexpressing triple negative breast cancer cells, inhibition of fatty acid oxidation reduces tumor growth [74]. Altering the balance of fatty acid synthesis and oxidation in tumors might be important to suppress cancer progression in a context-dependent manner. Additionally, the efficacy of different types of inhibitors for the lipid metabolism pathways on tumor suppression should be investigated in the future.

In vivo roles of p53-regulated, lipid metabolism-related proteins—SIRT1 and aromatase—in tumor progression have been shown using mouse models. Loss of one allele of *SIRT1* (*SIRT1^+/−^*) in mice accelerates tumor onset and incidence in the *p53^+/−^* background, indicating that *SIRT1* haplo-insufficiency facilities tumorigenesis [75], while overexpression of SIRT1, specifically in the gut, reduces the number and size of intestinal tumors in *APC^min/+^* mice [76]. McPherson et al. [77] show that *aromatase* knockout mice develop prostate hyperplasia. On the other hand, overexpression of aromatase in testis and the mammary gland increases the incidence of testicular cancer and mammary ductal adenocarcinomas, respectively [78,79]. Thus, in vivo roles of aromatase in tumor development could be cell type- and tissue contextdependent. Nonetheless, future studies testing in vivo roles of other p53 targets associated with lipid metabolism in tumorigenesis are needed to better understand contributions of these proteins to p53-mediated tumor suppression.

In summary, loss of p53 activity and mutations in p53 helps accelerate lipid accumulation, which could further contribute to cancer progression. Thus, lipid metabolism pathways can be targeted for cancer therapy, particularly for cancers lacking p53 or having p53 mutations.

## Figures and Tables

**Table 1:** Targets of wild-type p53 in lipid metabolism.

Targets	Effects of Wtp53	Biological Consequence	Reference
G6PD	Inhibit the activity by physical binding.	Loss of p53 activates G6PD and the pentose pathway, leading to lipid accumulation in the liver.	[16]
SREBP-1	Transcriptionally repress the expression.	Disruption of *p53* in *ob/ob* mice restores the expression of lipogenic enzymes regulated by SREBP-1.	[20]
SIRT1	A complex of p53 and Foxo3a transactivates SIRT1.	In *p53^−/−^* mice, nutrient starvation fails to increase SIRT1. It remains unclear whether increased lipid accumulation in *p53^−/−^* mice is due to attenuated SIRT1 levels.	[21]
Aromatase	Transcriptionally increase the expression.	*p53^−/−^* mice have lower levels of aromatase, resulting in higher levels of testosterone and lipid accumulation, which is nullified by transgenic expression of aromatase.	[22]
Acad11	Transcriptionally increase the expression.	Although Acad11 plays a key role in p53-mediated OXPHOS and cell survival upon glucose starvation, it is unclear whether increased Acad11 levels by p53 enhance fatty acid β-oxidation and how enhanced fatty acid β-oxidation contributes to cell survival.	[23]
Lipin1	Transcriptionally increase the expression.	Glucose restriction in C2C12 cells phosphorylates p53, leading to upregulation of Lipin1 and fatty acid oxidation.	[24]
MCD	Transcriptionally increase the expression.	*Mdm2^C305F^* mice show attenuated MCD induction and increased fatty acid accumulation in the liver under ribosomal stress, due to lack of inhibitory effects of RPs on Mdm2 and reduction in the p53 activity.	[25]
DHRS3	Transcriptionally increase the expression.	Activation of p53 upregulates DHRS3 which is associated with lipid droplets accumulation.	[26,27]
Caveolin 1	Transcriptionally increase the expression.	Overexpression of p53 upregulates Caveolin 1, leading to redution in intracellular free choleserol and viable cell growth.	[28]

**Table 2:** Targets of mutant p53 in lipid metabolism.

Targets	Effects of Mutp53	Biological Consequence	References
SREBPs	Bind and activate the transcription activity.	In breast cancer cells expressing mutp53, increased activities of SREBPs enhance the mevalonate pathway and accelerate growth in the 3D culture.	[58]
AMPK	Bind and inhibit the kinase activity.	GOF p53 mutants bind to and inhibit AMPK activity. It remains unclear how significantly the mutp53’s inhibitory effect on AMPK contributes to fatty acid synthesis and tumor progression.	[59]
